# Abnormal Levels of Metal Micronutrients and Autism Spectrum Disorder: A Perspective Review

**DOI:** 10.3389/fnmol.2020.586209

**Published:** 2020-12-10

**Authors:** Supriya Behl, Sunil Mehta, Mukesh K. Pandey

**Affiliations:** ^1^Department of Pediatric and Adolescent Medicine, Mayo Clinic, Rochester, MN, United States; ^2^Department of Radiology, Mayo Clinic, Rochester, MN, United States

**Keywords:** metal micronutrients, biometals, copper, iron, magnesium, zinc, selenium and autism spectrum disorder

## Abstract

The aim of the present review is to summarize the prevalence of abnormal levels of various metal micronutrients including copper (Cu), iron (Fe), magnesium (Mg), zinc (Zn), and selenium (Se) in Autism Spectrum Disorder (ASD) using hair, nail and serum samples. A correlation of selected abnormal metal ions with known neurodevelopmental processes using Gene Ontology (GO) term was also conducted. Data included in this review are derived from ASD clinical studies performed globally. Metal ion disparity data is also analyzed and discussed based on gender (Male/Female) to establish any gender dependent correlation. Finally, a rational perspective and possible path to better understand the role of metal micronutrients in ASD is suggested.

## Introduction

### Autism Spectrum Disorder: Epidemiology and Etiology

Autism spectrum disorder (ASD) is a complex condition characterized by impaired social communication and restricted, repetitive behaviors (Lord et al., 2018). Globally, it is estimated that 0.76% of children have ASD. The United States of America (USA) prevalence rates are higher at 1.9%, and have increased since 2000 (Maenner et al., 2020). The USA prevalence varies by race and ethnicity: prevalence in Hispanic children is approximately 17% lower than that of white children. Approximately one third of children with ASD also have an intellectual disability.

ASD is a heterogeneous condition which is likely caused by multiple environmental and genetic factors, and many risk factors have been identified. In a recent literature review of 67 environmental risk factors and 52 biomarkers, the most convincing risk factors for ASD were maternal factors before or during pregnancy. These included maternal age (≥35 years), maternal chronic and gestational hypertension, maternal overweight pre-pregnancy or during pregnancy, maternal pre-eclampsia, pre-pregnancy maternal antidepressant use (Kim et al., 2019), and maternal immune activation (Bilbo et al., 2018). The gut microbiome is also implicated in ASD symptomatology. In fact, gut microbiome plays an integral role in immune reactions and inflammation. One hypothesis is that various antigens induce peripheral immunoreactions through the GI tract, which subsequently alters CNS activity (Cristiano et al., 2018). Further, mouse models of maternal immune activation results in ASD-like behaviors as well as decreased gastrointestinal tract permeability. Because of this, diet modulations have been suggested as a therapeutic option for ASD (Rosenfeld, 2015). Other likely environmental risk factors for ASD include to paternal age (>45 years) (Gabis et al., 2010; Simard et al., 2019), and family history of autoimmune diseases (Wu et al., 2015) such as psoriasis (Croen et al., 2019), rheumatoid arthritis (Rom et al., 2018), placenta morphology (Straughen et al., 2017), and type 1 diabetes (Atladottir et al., 2009).

Since early twin studies in the late 1980’s, a strong genetic influence is seen for ASD. A recent literature review of all twin studies on ASD found a 0.98 correlation between monozygotic twins and an overall heritability estimate of approximately 64–91% (Tick et al., 2016). Today, over 900 autism susceptibility genes have been reported (Banerjee-Basu and Packer, 2010), indicating the condition’s heterogeneous nature. Both common single nucleotide polymorphisms (SNPs) and more rare copy number variations (CNVs) likely play a role in ASD incidence. A study looking at common SNPs across the genome found that ASD may be caused by a collective effort of many small effect SNPs rather than one or a few genes (Klei et al., 2012). CNVs in ASD are common on nine of 23 chromosomes (Bergbaum and Ogilvie, 2016), and have been reported on every chromosome. While most genetic variations are inherited in ASD, approximately 10% of ASD can be attributed to *de novo* genetic variations (Sebat et al., 2007).

## The Role of Metals in ASD

There is a growing interest in the role of metals in ASD incidence. The increasing environmental dissemination of toxic metals and chemicals are likely a cause for ASD, among other neurodevelopmental disorders (Grandjean and Landrigan, 2014). Typical ASD characteristics such as intellectual disability and language problems have been associated with exposure of toxic metals prior to or during pregnancy. In a review of almost 100 studies, 74% suggest that physiological mercury levels are a risk factor for ASD (Kern et al., 2016), likely due to autoimmune activation, oxidative stress, and subsequent neuroinflammation. Aluminum is found at high levels in both white and gray matter of ASD patients, and is hypothesized to be able to cross the blood-brain barriers and taken up by microglial cells (Mold et al., 2018). Further, animal studies examining neurodevelopmental effects of metals found changes in brain structures also implicated in ASD (Arora et al., 2017).

Aside from heavy and toxic metals, which actively disrupt neurodevelopmental processes, dyshomeostasis of metal micronutrients may also be involved in ASD etiology. Zinc, copper, selenium, iron, and magnesium levels have been associated with ASD incidence.

### Zinc (Zn)

Zinc is the second most abundant element in the human body and is involved in a plethora of cellular functions. Approximately 2,800 proteins or 10% of the human proteome may bind zinc *in vivo* (Andreini et al., 2006). Zinc is particularly involved in glutamatergic transmission (i.e., GABA pathway) during embryonic and childhood development. Deficiency of zinc in mice models leads to altered neural tube closure (Li et al., 2018) and ASD-related behavior such as weakened vocalization and social behavior through SHANK proteins, a family of postsynaptic scaffolding proteins (Grabrucker et al., 2014). When dietary zinc is added, non-ASD behavior is restored in SHANK3-mutant mice (Fourie et al., 2018). Brain zinc levels are 10-fold higher than serum Zinc levels, indicating a larger role of Zinc in neurodevelopment (Portbury and Adlard, 2017). Zinc is more abundant in neuronal-rich areas, and is important in neuronal modulation, synaptic plasticity, learning, and memory (Qi and Liu, 2019). Of note, current literature link zinc to ASD in the context of the central nervous system, and therefore a lot remains unknown within the context of the peripheral nervous system. Novel pharmacological therapies for brain injury target levels of free zinc in the brain to restore homeostasis, indicating the significance of zinc homeostasis in the brain (Frederickson et al., 2005).

Zinc is also involved in the gut-brain interaction, and many ASD patients also have gastrointestinal symptoms. Maternal zinc levels are likely a factor in fetal gut formation and therefore the gut-brain interaction in ASD (Vela et al., 2015). Prenatal zinc levels also influence the morphology of placenta (Wilson et al., 2017), and placental function is notable in ASD (Straughen et al., 2017).

There is a well-known link between autism and the immune system (Meltzer and Van de Water, 2017; Hughes et al., 2018), to which zinc plays an integral role in both innate and adaptive immunity, such as monocytes, natural killer, T-, and B-cells (Shankar and Prasad, 1998).

There is considerable evidence for an association between zinc deficiency and ASD (Yasuda and Tsutsui, 2013; Li et al., 2014; Goyal et al., 2019). Zinc-binding genes associated with ASD are up-regulated in all neurodevelopmental stages (Supplementary Table S1). In a study examining 1,967 children with ASD, almost 30% had low zinc concentration in hair samples (Yasuda et al., 2011). Another small study found lower zinc levels in saliva of autistic children when compared to healthy controls (Deshpande et al., 2019). Zinc levels may also be correlated to severity of ASD presentation (Guo et al., 2018). It is important to note, however, that significant variance is observed when comparing zinc from hair and nails (Giuseppe De Palma et al., 2011; Lakshmi Priya and Geetha, 2011), suggesting that serum may be a better source for zinc measurement. When serum was evaluated in 78 children with autism, 71.8% of children had zinc levels either in the lowest 10% or below the reference range (Faber et al., 2009).

Zinc levels may also be affected by geographic-specific factors (Table 1); studies in Ireland (Sweetman et al., 2019) and Brazil (Saldanha Tschinkel et al., 2018) found zinc levels in ASD children to be equivalent to that of healthy controls. One study in Oman found higher levels of zinc in ASD patients than in controls (Al-Farsi et al., 2013). Geographical differences may be attributed to differences in social determinants of health such as nutrition, economic status, and associated illnesses. Zinc deficiency in infants is prevalent in countries with malnourishment, and is globally a recognized public health issue (Ackland and Michalczyk, 2016). Geographic-specific differences may also be due to sample size and age variability between studies.

**TABLE 1:** Geographical differences in physiological zinc levels.

Country	Gender*^†^ (male: female)	Age in years*	Source	ASD mean zinc level	Control mean zinc level	*p*-value	References
**Africa**							
Egypt	3:1	4.1 ± 0.8	Hair	304.99 ± 25.8 μg/mg	419.5 ± 45.96 μg/mg	n/a	Eman Elsheshtawy et al., 2011
**Antarctica—N/A**							
**Asia**							
China	4:1	3.78 (SD 1.22)	Serum	78.7 (SD 7.0) μg/dl	87.7 (SD 8.7) μg/dl	<0.001	Li et al., 2014
India	4:1	4–12	Hair	130.46 (SD 15.65) μg/g	171.68 (SD 20.60) μg/g	<0.01	(Lakshmi Priya and Geetha, 2011)
			Nails	150.83 (SD 18.09) μg/g	193.98 (SD 23.27) μg/g	<0.01	Lakshmi Priya and Geetha, 2011
Japan	3.8:1	0–15	Hair	∼30% of ASD patients had a > 2 SD lower zinc concentration than reference	129 (range 86.3–193) ppm		Yasuda and Tsutsui, 2013
Oman	4.4:1	3–14 (mean 5.3)	Hair	Median 5.4 (quartile 0.82) μg/g	Median 2.9 (quartile 2.2) μg/g	0.0001	Al-Farsi et al., 2013
Saudi Arabia	5.3:1	3–9	Hair	67.04 (SD 23.78) mg/kg	110–227 mg/kg	n/a	Blaurock-Busch et al., 2012
**Australia**—**N/A**							
**Europe**							
Ireland (Northern)	7.2:1	2–18	Serum	11.68 (SD 1.7) μmol/L	11.63 (SD 2.1) μmol/L	0.86	Sweetman et al., 2019
Romania	Not reported	5.83 ± 3.10	Whole blood	5.54 (SD 0.78) μg/ml	6.14 (SD 0.76) μg/ml	0.005	Craciun et al., 2016
United Kingdom	3.8:1	2–16 (mean 7.0)	Serum	10.01 (SD 1.52) μmol/L	11.76 (SD 2.14) μmol/L	<0.001	Goyal et al., 2019
Italy	3:1	2–6	Serum	1021.52 (SD 100.76) ng/g	808.03 (SD 131.89) ng/g	0.01	Vergani Lauraad et al., 2011
	5.3:1	9.00 ± 4.05	Hair	Median 149 (25th to 75th percentile 89.00—187.75) μg/g	Median 143(25th to 75th percentile 105.50—166.35) μg/g	0.428	Giuseppe De Palma et al., 2011
Slovenia	7.8:1	1–16	Serum	10.74 (SD 1.81) μmol/L	12.10 (SD 1.52) μmol/L	0.007	Marta Macedoni-Lukšič et al., 2014
Russia	Not reported	2–9 (mean 5.12 ± 2.36)	Hair	Median 124.6 (25th to 75th percentile 77.0–174.2) μg/g	Median 113.3 (25th to 75th percentile 69.4–166.3) μg/g	0.365	Skalny et al., 2017
**North America**							
United States	3.5:1	6.3 (SD 3.67)	Serum	76.89 (SD 14.1) μg/dl	Reference 66 μg/dl	n/a	Faber et al., 2009
	6.2:1	11.7 ± 5.62	Serum	78.36 (SD 20.32) mg/dL	84.42 (SD 24.18) mg/dL	0.3541	Russo and Devito, 2011
Canada	4:1	3.90 ± 1.68	Red-cell	134.95 (SD 23.94) μmol/L	148.27 (SD17.09) μmol/L	0.08	Joan Jory, 2008
**South America**							
Brazil	Not reported	<18	Serum	105 (SD 0.73) mg/dl	Not reported.	n/a	Saldanha Tschinkel et al., 2018
Venezuela	Not reported	Not reported	Serum	180.50 ± 57.72 μg/dl	219.49 ± 72.10 μg/dl	N.S.	Semprún-Hernández et al., 2012

*For studies that list control and ASD patient age/gender separately, ASD patient information is reported in this table. ^†^Gender ratios were calculated from reported numbers for simplicity. SD denotes standard deviation and variance reported are as published in the literature. If variance was not specified as standard error or deviation, variance is reported as mean ± variance.

### Copper (Cu)

Copper also has important roles in the human body, and is involved in cell growth, among many others. Copper is involved in reactions connected to neurological diseases, and dyshomeostasis of copper has been seen in disorders such as Parkinson’s, Alzheimer’s, and Huntington’s Diseases (Faber et al., 2009). Further, copper is integral in several autism-related biological processes, such as immunity (Kelley et al., 1995) and placental development. Copper levels are typically higher than average in ASD patients.

In 78 children with ASD, 15.4% had higher copper levels than the reference range, and 30.8% were in the highest 10% of the copper reference range (Faber et al., 2009). In another study, mean serum copper levels were significantly higher in ASD children than in healthy controls (Li et al., 2014). A third study of 79 autistic individuals found a similar pattern, in which autistic and Pervasive Developmental Disorder-Not Otherwise Specified (PDD-NOS) patients had significantly higher plasma levels of copper (Russo and Devito, 2011). Increase in physiological copper levels may also correlate with increasing severity of ASD (Lakshmi Priya and Geetha, 2011). Copper levels in ASD patients do not seem to vary by geographical region (Table 2); regardless of region, copper levels vary in comparison to controls between studies.

**TABLE 2:** Geographical differences in physiological copper levels.

Country	Gender*^†^ (male: female)	Age in years*	Source	ASD mean copper level	Control mean copper level	*p*-value	References
**Africa**							
Egypt	3:1	4.1 ± 0.8	Hair	26.5 ± 1.9 μg/mg	19.1 ± 4.4 μg/mg	n/a	Eman Elsheshtawy et al., 2011
**Antarctica**					**N/A**		
**Asia**							
China	4:1	3.78 (SD 1.22)	Serum	129.9 (SD 13.1) μg/dl	121.2 (SD 11.3) μg/dl	<0.001	Li et al., 2014
India	4:1	4–12	Hair	36.62 (SD 4.39) μg/g	12.31 (SD 1.47) μg/g	<0.001	Lakshmi Priya and Geetha, 2011
			Nails	28.85 (SD 3.46) μg/g	9.62 (SD 1.15) μg/g	<0.001	
Oman	4.4:1	3–14 (mean 5.3)	Hair	Median 1.2 (quartile 0.1) μg/g	Median 6.6 (quartile 0.7) μg/g	0.02	Al-Farsi et al., 2013
Saudi Arabia	5.3:1	3–9	Hair	133.86 (SD 115.47) mg/kg	6.7–37 mg/kg	n/a	Blaurock-Busch et al., 2012
**Australia**					**N/A**		
**Europe**							
Romania	Not reported	5.83 ± 3.10	Whole blood	1.26 (SD 0.16) μg/ml	1.22 (SD 0.18) μg/ml	0.460	Craciun et al., 2016
Italy	3:1	2–6	Serum	1390.99 (SD 213.55) ng/g	1190.36 (SD 478.57) ng/g	N.S.	Vergani Lauraad et al., 2011
	5.3:1	9.00 ± 4.05	Hair	Median10.20 (25th to 75th percentile 8.12—13.00) μg/g	Median 9.40 (25th to 75th percentile 7.40–14.45) μg/g	0.374	Giuseppe De Palma et al., 2011
Slovenia	7.8:1	1–16 (mean 6.2 ± 3.0)	Serum	20.57 (SD 3.29) μmol/L	19.87 (SD 4.12) μmol/L	0.327	Marta Macedoni-Lukšič et al., 2014
Russia	Not reported	2–9 (mean 5.12 ± 2.36)	Hair	Median 10.2 (25th to 75th percentile 8.7–12.5) μg/g	Median 10.5 (25th to 75th percentile 9.2–12.6) μg/g	0.356	Skalny et al., 2017
**North America**							
USA	3.5:1	6.3 (SD 3.67)	Serum	129.68 (SD 29.1) μg/dl	Reference 153 μg/dl	n/a	Faber et al., 2009
	6.2:1	11.7 ± 5.62	Serum	111.50 (SD 27.73) mg/dL	90.42 (SD 19.55) mg/dL	0.0133	Russo and Devito, 2011
Canada	4:1	3.90 ± 1.68	Red-cell	14.38 (SD 1.39) μmol/L	14.90 (SD 2.29) μmol/L	0.22	Joan Jory, 2008
**South America**							
Brazil	Not reported	<18	Serum	83 (SD 0.37) mg/dl	Not reported.	n/a	Saldanha Tschinkel et al., 2018
Venezuela	Not reported	Not reported	Serum	128.62 ± 21.89 μg/dl	109.87 ± 24.65 μg/dl	<0.05	Semprún-Hernández et al., 2012

*For studies that list control and ASD patient age/gender separately, ASD patient information is reported in this table. ^†^Gender ratios were calculated from reported numbers for simplicity. SD denotes standard deviation and variance reported are as published in the literature. If variance was not specified as standard error or deviation, variance is reported as mean ± variance.

Interestingly, copper and zinc play competing roles physiologically, such that an increase in copper leads to zinc deficiency (Grabrucker, 2012). The zinc/copper ratio has therefore been examined in the ASD setting. Patients and children with ASD tend to have lower zinc/copper ratios than controls (Bjorklund, 2013), even if differences are not seen in copper levels alone (Craciun et al., 2016). Such differences are not known to be sex-dependent (Faber et al., 2009), though copper levels alone may differ by sex due to oral contraceptive use (Babic et al., 2013). One study found that the zinc/copper ratio could be used as a diagnostic biomarker (Li et al., 2014). Zinc/copper cycles may play a role in ASD occurrence, and the rhythmicity of these cycles can be used as a diagnostic tool to classify ASD (Curtin et al., 2018).

### Selenium (Se)

Selenium and selenium-dependent proteins are essential in brain development and managing oxidative damage in the brain, and it has been suggested that dyshomeostasis in selenium may be associated with ASD incidence (Raymond et al., 2014). In a recent literature review of 10 studies comparing hair trace element levels in ASD and controls, four of them found a significant difference in Selenium levels. However, two found a significant increase in Selenium levels in children with ASD, and two found a significant decrease (Tinkov et al., 2019). Another meta-analysis found no significant differences in mean hair or erythrocyte selenium concentrations among 12 studies (Saghazadeh et al., 2017). Though dyshomeostasis is likely involved in ASD incidence, the contradictory data indicate a need for a more comprehensive study evaluating Selenium levels in ASD patients (Anatoly et al., 2018).

### Iron (Fe)

Iron is the most abundant trace element in the body (Wood and Sperling, 2019). Iron deficiency anemia is a major health concern in both developed and developing countries and can result in inadequate cellular function at a young age (Bener et al., 2017). Iron is involved in several neurodevelopmental processes, such as transmitter synthesis, myelin production, and synaptogenesis, and deficiency leads to malfunction of these processes (Pivina et al., 2019). Subsequently, iron deficiency is associated with developmental delay (McCann and Ames, 2007) and likely in ASD. Deficiency of iron has been seen in ASD children when compared to controls (Bener et al., 2017). While one meta-analysis in 2017 found lower iron levels in ASD patients than controls (Saghazadeh et al., 2017), another meta-analysis in 2018 found no differences in peripheral iron levels in ASD children (Tseng et al., 2018). Iron deficiency may be associated with ASD symptoms and particularly correlates with severity of emotional and behavioral problems (Saghazadeh et al., 2017).

### Magnesium (Mg)

Magnesium is involved in basic cellular processes such as nucleic acid formation and energy metabolism. In neurodevelopment, magnesium regulates glutamate-activated channels in neuronal membranes, a process highly correlated with ASD pathogenesis (Saghazadeh et al., 2017). Magnesium has been seen as deficient in children with ASD (Lakshmi Priya and Geetha, 2011). In combination with vitamin B6, magnesium has been argued as a potential nutritional intervention for ASD. However, several systematic reviews from the late 1990’s to early 2000’s found no substantial evidence for magnesium and vitamin B6 as treatment for ASD (Karhu et al., 2019). Since then, not many studies have examined the role of magnesium in ASD. A more recent review, however, found a significant magnesium deficiency in ASD patients, and suggests monitoring of magnesium status in patients with ASD (Saghazadeh et al., 2017).

## Association Between Metal Micronutrients and Biological Processes

Iron, zinc, and copper have been considered as essential metal nutrients for neurodevelopment processes. Iron is required for the enzyme ribonucleotide reductase that regulates the central nervous system. Iron also plays a role in myelin synthesis (Prado and Dewey, 2014). Zinc is needed for cell division because of its role in DNA synthesis. Zinc is also required for modulation of postsynaptic plasticity, NMDA receptors for glutamate and inhibits GABA receptor activation (Prado and Dewey, 2014). For example, mouse models indicate alterations in zinc levels lead to differences in both synaptic plasticity and neurogenesis (Nam et al., 2017). When mothers are deprived of zinc early in pregnancy or after birth, rodent pups exhibit impaired DNA synthesis and improperly incorporate of thymine in brain DNA (Sandstead, 1985). Zinc deficiency in early development also results in morphologic brain defects including the hippocampus, a part of the brain most notably significant in working memory.

Along with folic acid and vitamin A, copper is needed for the formation of the neural plate and neural tube very early in the development (Prado and Dewey, 2014). Copper is also a cofactor of dopamine-β-hydroxylase, peptidyl-a-monooxygenase and many other enzymes which are involved in vital central nervous system processes (Lutsenko et al., 2010; Telianidis et al., 2013). Copper is particularly involved in neurotransmitter synthesis and neuromodulation (Scheiber et al., 2014). Subsequently, several neurodegenerative diseases associate with copper dyshomeostasis. Other essential trace metals, such as Mn, Mo, and trace elements such as Se ions also play a critical role in neurodevelopment. Metal ion contents also vary with age (Lutsenko et al., 2010; Xu et al., 2012). Deficiency or dyshomeostasis of any of these metal ions will affect the neurodevelopmental process and may not be corrected even after the repletion of these metal ions (Lutsenko et al., 2010). Considering the importance of metal ions as integral part of various metalloproteins and enzymes, perturbation of metal ions homeostasis either through dietary deficiencies or via genetic alterations in metalloproteins and enzymes can have detrimental effect on neurodevelopment. There are many zinc-binding genes within each neurodevelopmental pathway (Supplementary Table S1), providing an example of metal micronutrients’ role in neurodevelopment.

## Sex and Gender Differences in ASD

ASD incidence is four times higher in males than in females (Baird et al., 2006; Baio et al., 2018), and ASD phenotypes present differently between sexes (Grove et al., 2017). Females may possess a protective factor for ASD, due to their earlier development of language (Dickerson et al., 2017). However, among individuals with a high intellectual capacity, females out-number males by a ratio of 11:1. When ASD patients do not have physical or cerebral abnormalities, this ratio has been seen at as high as 23:1 (Schaafsma and Pfaff, 2014).

ASD sex differences can be explained by many mechanisms. As ASD has a genetic component, there may be ASD-associated genes on the X and Y sex chromosomes. In a 2014 study examining children with an extra X chromosome (i.e., Klinefelter syndrome and Trisomy X), levels of social dysfunction and autism symptoms were higher than those of controls. Social anxiety in children with an extra X chromosome was at a higher level than ASD patients (van Rijn et al., 2014). Genes on each sex chromosome can be up- or down-regulated through a number of cellular mechanisms, all which may impact gene expression.

ASD differences may also be explained by hormonal mechanisms. One study found a significant difference in hormone levels between ASD patients and controls (Geier and Geier, 2006). Further, genes relating to sex steroids are associated with autistic phenotypes (Chakrabarti et al., 2009). Hormonal dyshomeostasis leading to ASD-like symptoms may be due to prenatal gonadal hormones: Prenatal and amniotic testosterone has been seen to correlate with ASD-similar behaviors, in both females and males (Schaafsma and Pfaff, 2014).

Previous studies show that levels of metal micronutrients differ by sex. For instance, copper levels are increased in women using oral contraceptives (Babic et al., 2013). The testosterone to estrogen conversion is regulated by a zinc-dependent protein. Zinc deficiency may therefore affect young girls and boys differently (Grabrucker et al., 2016). In a recent literature review of metal levels in ASD, a strong argument was made for the need to stratify results by sex. Many studies had been published on a plethora of metals and markers of exposure in hair, urine, blood, teeth, fingernails, and air pollution. However, only three studies were found to have reported on sex differences in metal levels. Further, results for each metal were conflicting; suggesting that stratification for sex in these studies may lead to more accurate findings. In the present review, all studies had a higher number of boys than girls studied, which is representative of the overall ASD population. Only one study evaluated sex differences in metal levels. Three studies did not report the gender distribution of their cohort. There are sex differences in metal exposures, metabolism, and physiological storage, which may explain possible metal differences between sexes (Faber et al., 2009; Dickerson et al., 2017).

## Perspective

There is a growing interest in the role of metal micronutrients in ASD. Metal micronutrients, essential to a large majority of biological processes, are recommended as therapeutic options for children with ASD (Cristiano et al., 2018). Particularly, zinc and copper, including the zinc: copper ratio, are evidently involved in ASD incidence and severity. The role of other metals, such as selenium, in ASD is less clear. Selenium is indeed crucial to neurological function, and it is implicated in neurological disorders (Pillai et al., 2014; Dominiak et al., 2016) such as Alzheimer’s (Solovyev et al., 2018), suggesting that it may also play a role in ASD. There is, however, conflicting evidence indicating the presence or absence of selenium in ASD patients. There remains a need for a more comprehensive look at selenium levels in ASD patients.

Many studies examining metal micronutrient levels in ASD patients did so with nail or hair trace levels, which is an arguable source for such analyses. Very few have compared hair, nail, and plasma levels of metal micronutrients. We recommend further investigations of the accuracy and reliability of using each of these sources in the ASD setting.

Finally, we recommend and encourage future analyses of metal micronutrients in ASD to be stratified by sex and age. ASD prevalence rates greatly differ by sex, and several hypotheses have been made to explain such differences. However, only three studies have been identified to stratify their results by sex when examining metals in ASD. Two of these three studies found significant sex differences in metal concentrations. Because of the inherent sex differences in ASD incidence and presentation, it is evident that there is a need for sex stratification in metal analyses in ASD. Further, age ranges should be considered when designing future studies regarding metal micronutrients in ASD. Throughout the course of the lifespan, clinical requirements differ (current clinical reference ranges are indicated in Supplementary Table S3). Therefore, future studies should delineate age brackets of participants.

Based on the summarized findings on the role of metal micronutrients in ASD, we also recommend analyzing the metal micronutrients in all diagnosed ASD children to broaden the understanding of association of metal micronutrients in ASD. Future studies should be conducted comprehensively, as small sample sizes have led to inconsistent results in the literature.

## Conclusion

Autism spectrum disorder (ASD) is a complex condition with a combination of both genetic and environmental factors involved in each case. While toxic metals and ASD incidence are widely characterized, much is to be understood of metal micronutrients in ASD. Zinc and copper play evident roles in neurodevelopment. The extents to which they play roles in ASD likely differ by geographic location. Of all genes involved in ASD incidence, 134 are zinc-binding. Within these, several ASD-related and metal-binding genes can be mapped to specific neurodevelopmental processes through a GO analysis.

There is a lot that is still unknown regarding metal micronutrients and ASD. Specifically, while selenium clearly plays a role in neurodevelopment, the literature shows conflicting evidence for selenium in ASD incidence. Further, studies often use one of hair, nail, or plasma as their source to compare concentrations, but clear differences have been seen in the three, suggesting comprehensive studies with multiple sources. Last, there remains a critical need to stratify metal micronutrient results by sex and age, considering the significant sex and age differences in all other aspects of ASD.

## Data Availability Statement

The original contributions presented in the study are included in the article/Supplementary Materials, further inquiries can be directed to the corresponding author/s.

## Author Contributions

SB wrote the review article under the guidance of MP and SM. MP and SM reviewed and edited the article. All authors have actively participated in completion of this manuscript.

## Conflict of Interest

The authors declare that the research was conducted in the absence of any commercial or financial relationships that could be construed as a potential conflict of interest.
