# Supplementary material for: Abnormal Levels of Metal Micronutrients and Autism Spectrum Disorder: A Perspective Review
Source: Front Mol Neurosci. 2020 Dec 10;13:586209. doi: 10.3389/fnmol.2020.586209 (PMC7759187; doi:10.3389/fnmol.2020.586209)
Supplement: Supplementary file 1 [file Table_1.pdf]

Supplementary Table S1: Neurodevelopmental processes likely indicated in ASD etiology for which zinc plays a role in. Genes associated with ASD were extracted from SFARI Gene (https://gene.sfari.org/) on March 4th, 2020. Genes were then inputted into UniProt's Retrieve/ID mapping tool to obtain UniProtKB identifiers. With UniProtKB identifiers, genes were then filtered for zinc finger motifs. The remaining 125 genes were inputted into a Gene Ontology analysis for biological processes with the GO Ontology database Released 2020-02-2, using a PANTHER Overrepresentation Test (significant p value of 0.05). Full gene list used in analysis is found in Supplementary Table 2.

Neurodevelopmental GO Terms	Number of zinc-binding ASD Genes upregulated in process (N =125)	Fold enrichment	Raw p-value	Sources (Pubmed ID)
**Stem cell proliferation**				28770438
positive regulation of epithelial cell proliferation (GO:0050679)	8	7.19	2.10E-05	
regulation of epithelial cell proliferation (GO:0050678)	11	5.7	4.93E-06	
negative regulation of nervous system development (GO:0051961)	8	4.46	5.08E-04	
positive regulation of nervous system development (GO:0051962)	12	3.79	9.31E-05	
regulation of nervous system development (GO:0051960)	19	3.51	2.14E-06	
negative regulation of cell differentiation (GO:0045596)	14	3.44	6.65E-05	
regulation of cell differentiation (GO:0045595)	36	3.42	3.26E-11	
negative regulation of developmental process (GO:0051093)	16	2.91	1.36E-04	
positive regulation of developmental process (GO:0051094)	23	2.9	3.84E-06	
regulation of developmental process (GO:0050793)	42	2.8	2.33E-10	
nervous system development (GO:0007399)	37	2.72	8.97E-09	
cell differentiation (GO:0030154)	42	1.97	6.18E-06	
developmental process (GO:0032502)	66	1.96	6.37E-10	
**Cell fate specification**				28770438
positive regulation of neuron projection development (GO:0010976)	10	5.98	9.08E-06	
positive regulation of neuron differentiation (GO:0045666)	12	5.5	2.55E-06	
regulation of neuron differentiation (GO:0045664)	17	4.45	3.43E-07	
negative regulation of neurogenesis (GO:0050768)	8	4.8	3.18E-04	
positive regulation of neurogenesis (GO:0050769)	12	4.32	2.73E-05	
regulation of neurogenesis (GO:0050767)	19	3.97	3.52E-07	
generation of neurons (GO:0048699)	26	2.92	7.11E-07	
neurogenesis (GO:0022008)	26	2.74	2.31E-06	
**Neuronal migration**				3897914
Positive regulation of endothelial cell chemotaxis by VEGF-activated vascular endothelial growth factor receptor signaling pathway (GO:0038033)	2	70.09	6.58E-04	
regulation of neuron projection development (GO:0010975)	14	4.76	1.86E-06	
**Synaptic genesis/plasticity**				3897914
Regulation of dendrite extension (GO:1903859)	4	28.03	2.10E-05	
positive regulation of dendrite extension (GO:1903861)	3	22.85	4.21E-04	
**Neurotransmitter release**				
calcium ion-regulated exocytosis of neurotransmitter (GO:0048791)	3	23.89	3.74E-04	
regulation of neurotransmitter secretion (GO:0046928)	5	7.96	5.05E-04	

Behl S, Mehta S and Pandey MK (2020) Abnormal Levels of Metal Micronutrients and Autism Spectrum Disorder: A Perspective Review. Front. Mol. Neurosci. 13:586209. doi: 10.3389/fnmol.2020.586209
