# Supplementary material for: Abnormal Levels of Metal Micronutrients and Autism Spectrum Disorder: A Perspective Review
Source: Front Mol Neurosci. 2020 Dec 10;13:586209. doi: 10.3389/fnmol.2020.586209 (PMC7759187; doi:10.3389/fnmol.2020.586209)
Supplement: Supplementary file 3 [file Table_3.pdf]

Supplementary Table S3. Clinical reference ranges for selenium, copper, and zinc used in the United States.

Age	Selenium<br>ng/mL	Copper<br>mcg/mL	Zinc<br>mcg/mL
0 – 2 Months	45-90	0.40-1.40	0.60-1.20
3 – 6 Months	50-120	0.40-1.60	0.60-1.20
7 – 9 Months	60-120	0.40-1.70	0.60-1.20
10 – 12 Months	70-130	0.80-1.70	0.60-1.20
13 Months – 10 Years	70-150	0.80-1.80	0.60-1.20
> 11 Years	70-150	0.75-1.45	0.66-1.10
